# Advanced Polymer Composites and Applications

**DOI:** 10.3390/polym17152062

**Published:** 2025-07-28

**Authors:** Bing Wang, Lihua Zhan, Chenglong Guan

**Affiliations:** 1 Fujian Provincial Key Laboratory of Terahertz Functional Devices and Intelligent Sensing, School of Mechanical Engineering and Automation, Fuzhou University, Fuzhou 350108, China; b.wang@fzu.edu.cn; 2 State Key Laboratory of Precision Manufacturing for Extreme Service Performance, Central South University, Changsha 410083, China; yjs-cast@csu.edu.cn; 3 Light Alloys Research Institute, Central South University, Changsha 410083, China

Polymer composite materials, engineered by combining polymeric matrices with functional fillers or reinforcing phases, represent a frontier in advanced materials science driven by the dual imperatives of performance enhancement and sustainable development. The production of polymer matrix composite components encompasses a multifaceted process involving structural design, preform shaping, curing and forming, machining and assembly, and inspection and analysis. It is imperative to recognize that each of these steps has a direct and substantial impact on the overall performance and functionality of the resultant structure. In light of the escalating adoption of polymer and composite materials in high-end applications across various industries, composite components are evolving towards larger scale, integral structures, increased complexity, and multi-functions. These developments inevitably give rise to heightened challenges in the design and manufacturing of advanced functional polymers and composites.

In this Special Issue, we have collected the most recent advances in functional polymers and composites, including 10 original research papers and two review articles, co-authored by 71 scientists and engineers from 35 institutions and 10 countries. The research topics mainly covered the development of functional polymers, structural design, molding processes, and engineering applications.

With the increasingly widespread application of advanced polymer composites in aerospace, satellite communications, automobiles, and other fields, higher demands are now placed on their functionalities—such as electromagnetic shielding performance and thermal insulation performance—while ensuring that the materials possess excellent fundamental mechanical properties. The development of nanocomposites based on filler modification technology provides a solution for achieving functional integration in these materials. Khalil et al. [1] synthesized Zn-Al ferrite/polypyrrole nanocomposites and conducted a comprehensive characterization. X-ray diffraction analysis confirmed the presence of ZnO, AlFeO_3_, and Fe_2_O_3_ phases, and high-resolution transmission electron microscopy (HR-TEM) revealed a distinctive core–shell morphology. Variations in aluminum content not only affect magnetization, but also alter the dielectric permittivity and relaxation time, thus demonstrating that the Zn-Al ferrite/polypyrrole nanocomposite is a promising candidate material for advanced microwave absorption applications. Li et al. [2] proposed an effective and universal filler modification and nanocomposite preparation method. Specifically, the montmorillonite (MMT) surface was coated with polydopamine (PDA) to obtain DMMT, and then DMMT gel was compounded with solid ethylene propylene diene monomer rubber (EPDM) via the gel compounding method. Compared with the unmodified MMT filler EPDM, the EPDM/DMMT nanocomposite showed much fewer filler aggregates in the matrix, and the highest tensile strength of the composites reached 6.5 MPa with 10 phr DMMT, almost 200% higher than that of pure EPDM.

Moreover, attributes such as low cost, recyclability, and environmental friendliness also constitute crucial factors that need to be considered during the R&D process of novel polymers and composites. Jasinski et al. [3] explored the possibility of the utilization of coniferous bark as a filler in wood–polymer composites (WPCs), and discovered that bark-filled composites exhibit lower thickness swelling and water absorption, as well as lower water contact angles and surface free energy, compared to analogous composites filled with coniferous sawdust. Ail et al. [4] aimed to utilize waste black tea bags (BTBs) and date palm surface fibers (DPSFs) efficiently by developing new composite materials for thermal insulation and sound absorption. Hybrid boards prepared by bonding BTBs and DPSFs with polyvinyl acetate resin were tested and found to possess favorable thermal conductivity, noise reduction performance, and thermal stability, along with high mechanical properties. This material not only shows potential for competing with synthetic counterparts derived from petrochemicals in building insulation, but also helps reduce the number of landfills and the level of environmental pollution.

The development of novel material systems forms the foundation for achieving multifunctional integration, while geometric structure design and advanced manufacturing techniques are core to ensuring both dynamic and static mechanical properties, along with the forming quality, of polymer matrix composite components; they are also pivotal for determining whether these components can achieve practical engineering application. Huang et al. [5] investigated the shrinkage behavior of core height in honeycomb sandwich structures during the secondary bonding process and found that the out-of-plane compression deformation of aramid honeycomb cores was primarily caused by dehumidification, pressurization, and creep. By employing the viscoelastic Burgers mechanical model and applying the nonlinear surface fitting method, the total height shrinkage deformation behavior of the aramid honeycomb core during the curing process can be accurately predicted. Addressing the critical role of bone scaffolds in tissue engineering, Bakhtiari et al. [6] fabricated four types of polylactic acid (PLA) scaffolds with distinct structures via 3D printing. They investigated the influence of pore architecture on the scaffolds’ mechanical properties under quasi-static and cyclic compression, revealing that increased strut thickness correlated with higher compressive strength, while enhanced fatigue performance across different topologies was associated with the minimum cross-sectional area of the scaffolds. However, fatigue damage was observed in all structures under higher strains. Zhang et al. [7] investigated the influence of vibration parameters on the porosity, fiber weight fraction, and mechanical properties of polymer composite components fabricated via the vibration pretreatment–microwave curing process. Utilizing an orthogonal experimental design to optimize the process parameters, they found that vibration acceleration alters the escape pathways of pores within the components, which is the dominant factor affecting their mechanical properties. Furthermore, a positive correlation was demonstrated between the interlaminar shear strength and impact strength of the components.

It is precisely the development of these novel material systems, coupled with the introduction of new structures and processes, that have enabled polymer matrix composites to not only maintain their intrinsic attributes—such as high mechanical strength, low density, and processability—but also integrate functional properties including corrosion resistance, friction resistance, electromagnetic shielding, thermal insulation, and freeze resistance. This has significantly expanded their applications in traditional fields like aerospace, marine, and automotive engineering, while demonstrating promising potential in emerging domains such as healthcare, food processing, and construction. Barros et al. [8] produced recycled substrates via hot compression molding with different proportions of pure CuO and sludge incorporated onto surfaces, enhancing their antibacterial properties through surface functionalization. This approach not only enables the recycling of abandoned fishing nets into novel substrates for algae cultivation, but also offers a viable pathway for reusing sludges typically disposed of in landfills.

In the context of the biomedical applications of polymer matrix composites, Wilk-Kozubek et al. [9] delve into the intriguing phenomenon of low-temperature thermochromism, with a particular focus on its applications in temperature-sensitive fields like medical storage. This study highlights recent advances in polydiacetylenes (PDAs)—a class of conjugated polymers engineered through structural alterations of monomers to achieve irreversible color transitions at specific low temperatures—and identifies them as ideal materials for constructing reliable temperature indicators that ensure the integrity of thermally sensitive products. Giuliani et al. [10] developed electrospun composites based on polycaprolactone (polymer matrix) and tungsten powder for radiation shielding applications in high-dose environments such as aerospace and healthcare. They demonstrated that the radiation shielding effectiveness of these composites increased with thickness and/or the number of stacked layers, effectively reducing radiation-induced apoptosis.

In traditional industrial sectors such as construction and machinery, polymer coatings emerge as a more efficient and less wasteful alternative when considering component durability, manufacturing costs, and environmental sustainability—particularly through the use of adhesive polymers to ensure coating durability and functional effectiveness. Torskaya et al. [11] applied thin (several hundred microns thick) UHMWPE coatings to formed rubber rings and conducted friction tests on both the coated samples and pure UHMWPE under varying loads and velocities. Their results demonstrated that this polymer combination imparts antifrictional properties and wear resistance to the surface layer while preserving the damping performance of the rubber substrate. Kolya et al. [12] provided a comprehensive examination of eco-friendly polymer nanocomposite coatings, including their synthesis, characterization, and performance. By integrating nanoparticles—such as nano-clays, graphene oxide, and metal oxides—into biopolymer matrices, these nanocomposites demonstrate improved thermal stability and char formation properties. This significantly mitigates the flammability of wood substrates, supporting the expanded utilization of wood in sustainable construction practices and aligning with global initiatives toward achieving carbon neutrality.

We hope that this Special Issue will contribute to disseminating the latest progress in functional polymers and composites, as well as stimulating the interest of its audiences to work in this important and vibrant area to promote and benefit the multidisciplinary scientific communities. Owing to the word limit on this Editorial, audiences are recommended to refer to the original papers for further information on their specific interests.
